# Aryl hydrocarbon receptor activation mediates kidney disease and renal cell carcinoma

**DOI:** 10.1186/s12967-019-2054-5

**Published:** 2019-09-05

**Authors:** Hui Zhao, Lin Chen, Tian Yang, Ya-Long Feng, Nosratola D. Vaziri, Bao-Li Liu, Qing-Quan Liu, Yan Guo, Ying-Yong Zhao

**Affiliations:** 1 Faculty of Life Science & Medicine, Northwest University, No. 229 Taibai North Road, Xi’an, 710069 Shaanxi China; 2 Division of Nephrology and Hypertension, School of Medicine, University of California Irvine, Irvine, CA 92897 USA; 3 Beijing Hospital of Traditional Chinese Medicine, Capital Medical University, Beijing, 100010 China; 4 Department of Internal Medicine, University of New Mexico, Albuquerque, 87131 USA

**Keywords:** Aryl hydrocarbon receptor, Chronic kidney disease, Gut microbiota, Uremic toxins, Renal cell carcinoma, Natural products

## Abstract

The aryl hydrocarbon receptor (AhR) is a well-known ligand-activated cytoplasmic transcription factor that contributes to cellular responses against environmental toxins and carcinogens. AhR is activated by a range of structurally diverse compounds from the environment, microbiome, natural products, and host metabolism, suggesting that AhR possesses a rather promiscuous ligand binding site. Increasing studies have indicated that AhR can be activated by a variety of endogenous ligands and induce the expression of a battery of genes. AhR regulates a variety of physiopathological events, including cell proliferation, differentiation, apoptosis, adhesion and migration. These new roles have expanded our understanding of the AhR signalling pathways and endogenous metabolites interacting with AhR under homeostatic and pathological conditions. Recent studies have demonstrated that AhR is linked to cardiovascular disease (CVD), chronic kidney disease (CKD) and renal cell carcinoma (RCC). In this review, we summarize gut microbiota-derived ligands inducing AhR activity in patients with CKD, CVD, diabetic nephropathy and RCC that may provide a new diagnostic and prognostic approach for complex renal damage. We further highlight polyphenols from natural products as AhR agonists or antagonists that regulate AhR activity. A better understanding of structurally diverse polyphenols and AhR biological activities would allow us to illuminate their molecular mechanism and discover potential therapeutic strategies targeting AhR activation.

## Background

The induction of a battery of genes encoding xenobiotic metabolizing enzymes in response to chemical damage is an adaptive response in many organisms. The aryl hydrocarbon receptor (AhR) is a mediator of the toxic response of ubiquitous environmental pollutants such as halogenated aromatic hydrocarbons, polycyclic aromatic hydrocarbons and coplanar polychlorinated biphenyls [1–3], including 2,3,7,8-tetrachlorodibenzo-*p*-dioxin (TCDD), which has carcinogenic and teratogenic effects [4]. AhR is described as an environment-sensor period-aryl hydrocarbon receptor nuclear translocator-single minded (Per-ARNT-Sim) protein that belongs to a member of the family of basic helix-loop-helix transcription factors [5].

### AhR signalling and its ligands

#### AhR signalling

**Fig. 1:**
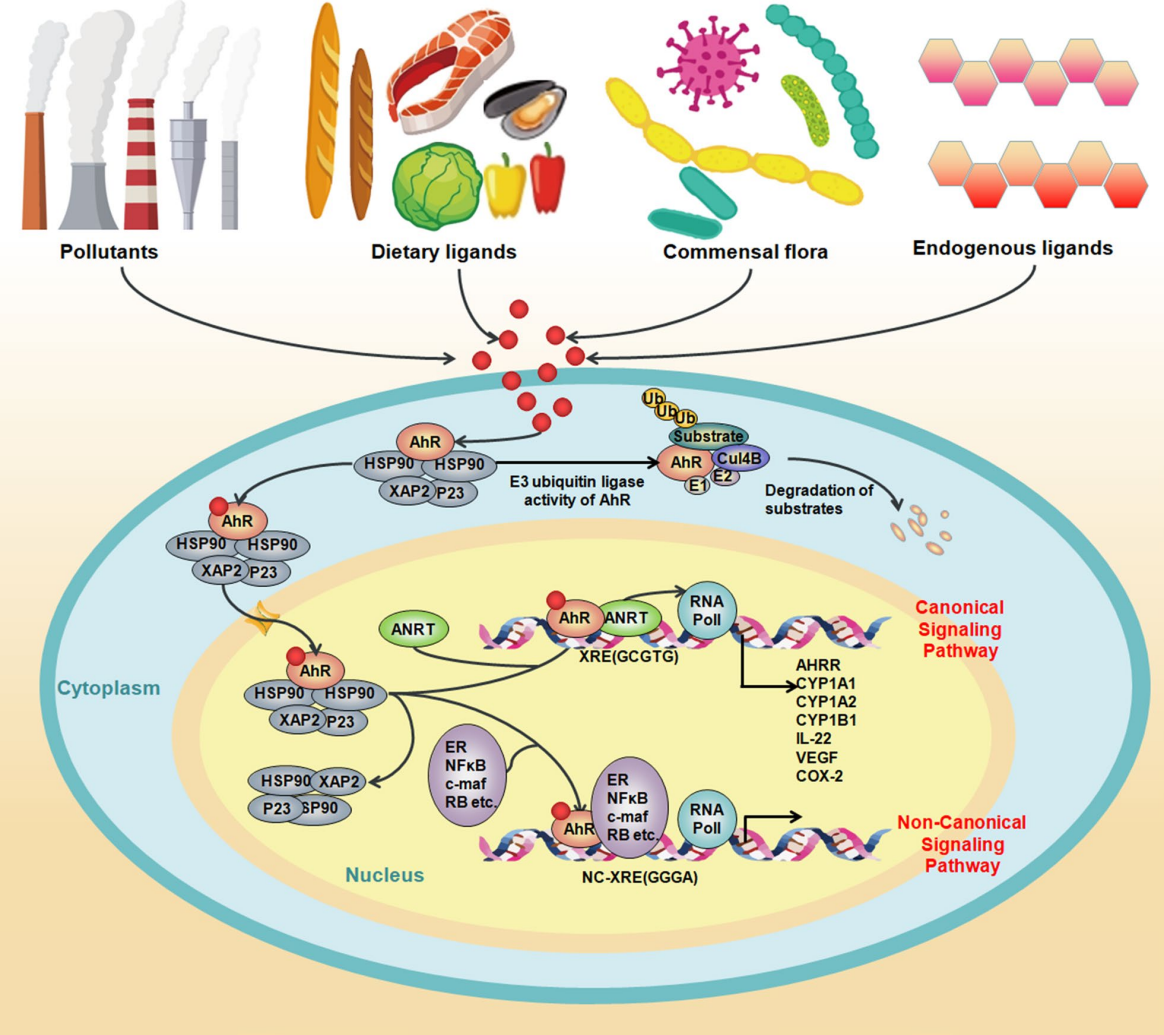
AhR transcription in mammalian cells and the putative mechanism of AhR activation. The inactive form of AhR occurs in the cytoplasm as a complex with chaperone proteins, including HSP90, P23 and XAP2. Multiple exogenous and endogenous AhR ligands from the environment, diet, host metabolism and gut microbiome induce a conformational alteration in AhR, exposing the nuclear localization signal to activate nuclear shuttling. Once in the nucleus, AhR forms a heterodimeric complex, with ARNT binding to the XRE sequence motif 5′-GCGTG-3′. This induces the expression of its target genes, such as *CYP1A1*, *CYP1A2*, *CYP1B1* and *COX*-*2*, which are involved in the inflammatory response and xenobiotic metabolism. Furthermore, AhR mediates AhR repressor expression, abrogating the formation of the AhR/ARNT heterodimer and inhibiting its transcriptional activity. Moreover, AhR forms as a Cul4B-based E3 ubiquitin ligase complex, inducing selective protein degradation. AhR regulation signalling can be controlled via nuclear export and subsequent AhR degradation through the ubiquitin–proteasome signalling pathway. In addition to this canonical pathway, signalling through AhR can also be mediated through interactions with other regulatory proteins, such as oestrogen receptor, NF-κB and RB

AhR is a ligand-mediated transcription factor implicated in the biological detoxification of ligands [6]. As shown in Fig. 1, under basal conditions, AhR is located in the cytoplasm in an inactive state as part of a complex formed with stabilizer proteins, including 2 molecules of heat shock protein 90 (HSP90), one molecule of cochaperone p23 (P23) and one molecule of X-associated protein 2 (XAP2) [1]. When a ligand binds to AhR, the AhR/ligand/Hsp90/XAP2 complex translocates into the nucleus and dimerizes with AhR nuclear translocator (ARNT). AhR is activated by a conformational alteration that exposes its nuclear localization sequence. After AhR is phosphorylated by protein kinase C, the AhR complex is translocated into the nucleus [7]. In the nucleus, the complex releases the protein so that it can bind to the ARNT through its Per-ARNT-Sim domain, leading to the AhR-ARNT dimer. This AhR/ARNT heterodimer is recognized by a DNA-specific site, 5′-GCGTG-3′, the DRE or XRE (dioxin- or xenobiotic-responsive element) sequence located within the promoters of target genes, and triggers their transcription, such as cytochrome P450, family 1, member 1A (*CYP1A1*); cytochrome P450, family 1, member 2A (*CYP1A2*); cytochrome P450, family 1, sub family B (*CYP1B1*); AhR repressor (*AhRR*); and cyclooxygenase-2 (*COX*-*2*). AhR induces the expression of xenobiotic enzymes, such as cytochrome P450 genes, needed for the detoxication of AhR toxic ligands [1].

**Fig. 2:**
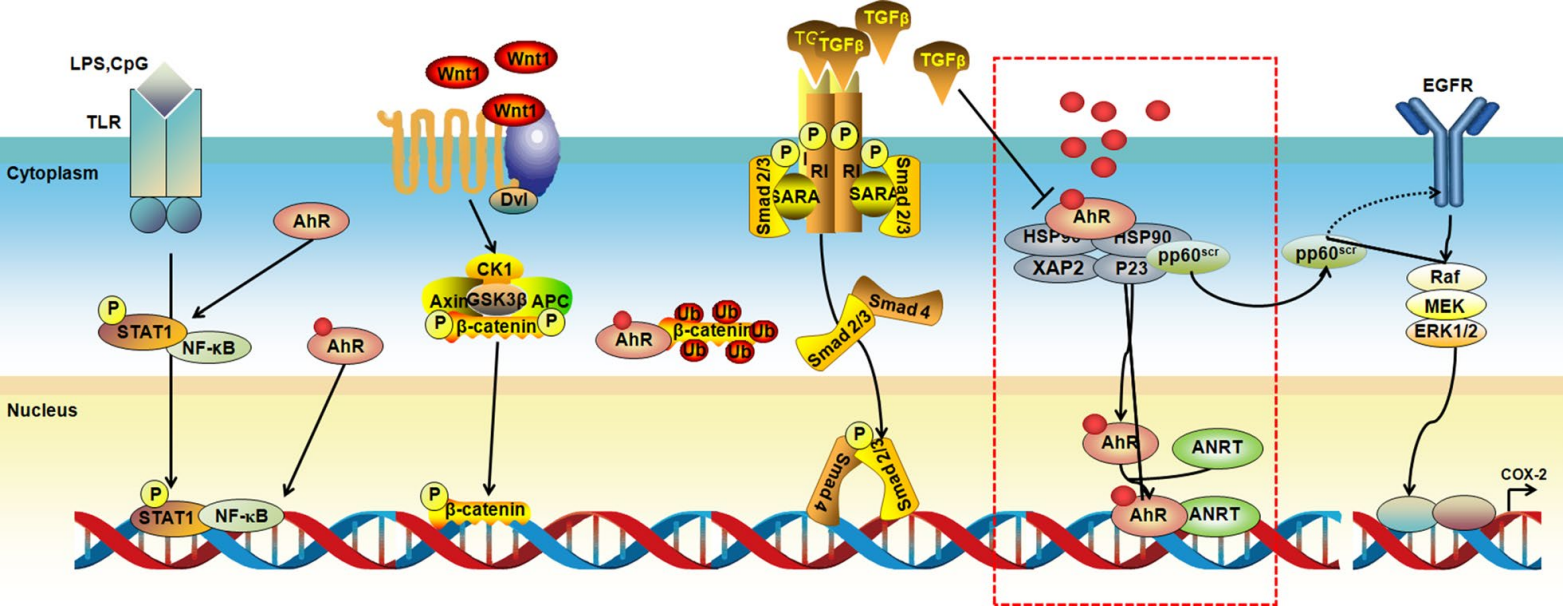
AhR interacts with multiple other signalling pathways. AhR activates other cytosolic proteins, including β-catenin, Smads, ERK, p38MAPK and JNK

AhR activation is primarily recognized to mediate the expression of phase I and phase II drug metabolism genes, including *CYP1A1*, *CYP1A2*, *CYP1B1*, *UGT1A1/6* and sulfotransferase *(SULT)1A1*. Mounting studies have demonstrated that the AhR pathway is associated with diverse physiological functions and disease processes, such as the regulation of T-cell differentiation and embryonic/foetal development, the mediation of oxidative stress and inflammatory responses [8–13]. In fact, traditional AhR signalling cannot explain all the cellular functions attributed to AhR. In addition to the canonical gene regulation pathway, noncanonical AhR signalling has been described that includes crosstalk with other transcription factors, including nuclear factor kappa B (NF-κB), nuclear factor-erythroid-2-related factor 2 (Nrf2), programmed death ligand 1 and activator protein 1 (notably the RelA subunit), hypophosphorylated retinoblastoma protein, the corepressor oestrogen receptor and the progesterone receptor [14–16] (Figs. 1 and 2). Furthermore, cytosolic AhR can activate a myriad of other cytosolic proteins, including β-catenin, Smads, mitogen-activated protein kinase (MAPK) family p38, extracellular signal-regulated kinase (ERK) and Jun-NH2-terminal kinase (JNK) [17] (Fig. 2).

#### Ligands of activated AhR signalling

There is emerging evidence that chronic exposure to environmental chemicals via air and diet, particularly persistent organic compound pollutants such as TCDD or dioxin, causes side effects by ligand-activated induction of the AhR pathway [18–20] (Fig. 1). Moreover, there are also a myriad of endogenous AhR ligand candidates, such as eicosanoids (e.g., lipoxin A4, bilirubin, and lipopolysaccharides), and a myriad of naturally occurring flavonoids (e.g., resveratrol and quercetin). These endogenous metabolites have been identified as weak AhR ligands due to their low affinity for AhR (e.g., bilirubin and indirubin). However, bilirubin can activate AhR at a certain concentration in certain disease states, such as jaundice [21]. Human AhR preferentially binds to indirubin compared to mouse AhR [22].

#### AhR activation through low-molecular-weight uremic toxins

Metabonomics, which has been defined as “the quantitative measurement of the dynamic multiparametric metabolic response of living organisms to physiopathological stimulation or genetic modifications” [23, 24], is used as a comprehensive method to address changes in low-molecular-weight metabolites (MW < 1000 Da) following disease, toxic exposure or variation in genetic function [25–28]. Mounting studies by using burgeoning metabonomics have demonstrated that low-molecular-weight metabolites, such as cholesterols, amino acids, vitamins, lipids, carbohydrates, minerals and other compounds, play a critical role in health and diseases [29–33]. A number of novel or known metabolites have been used for disease diagnosis and prognosis, new drug discovery and toxicity evaluation [34–41].

**Fig. 3:**
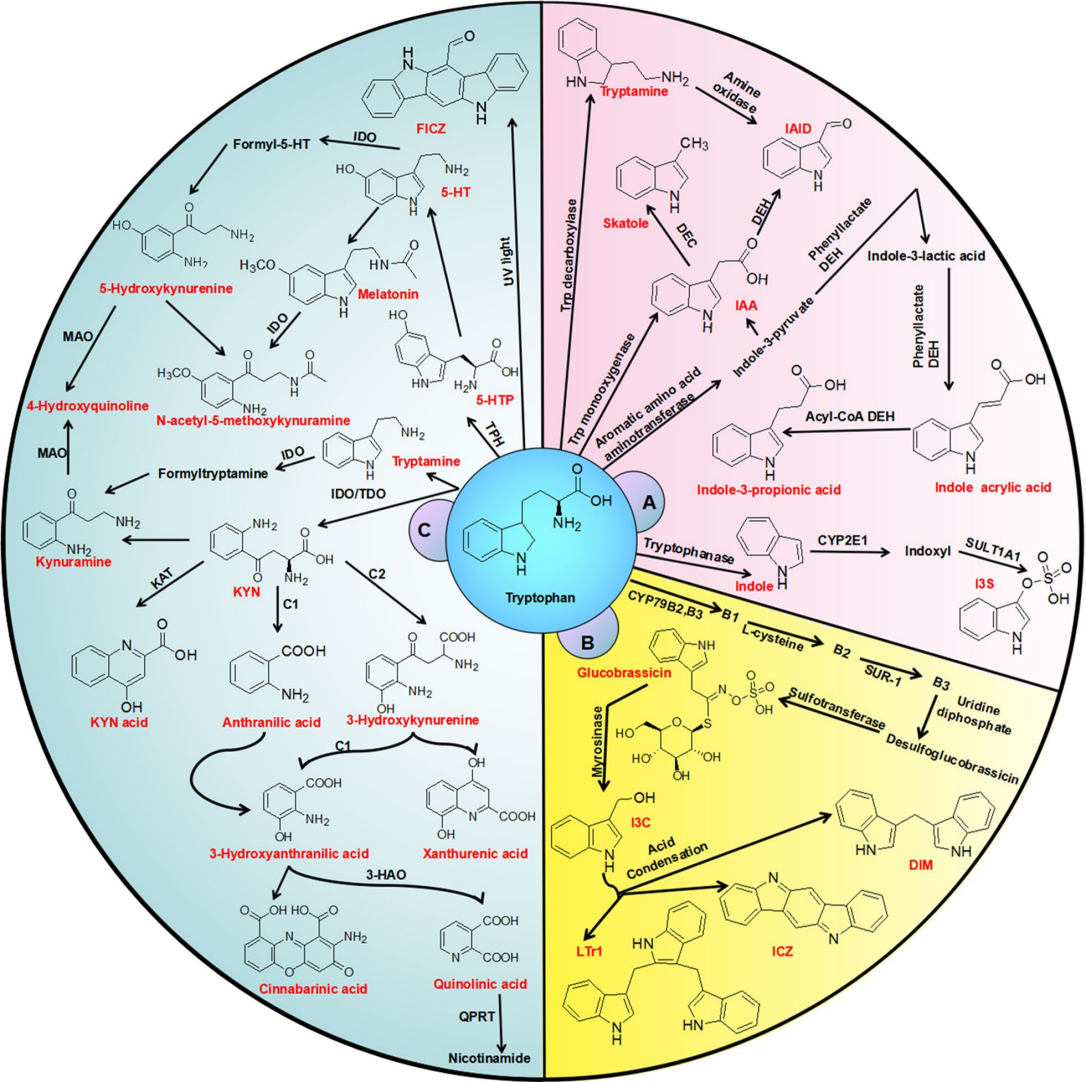
Biosynthesis of AhR ligands from tryptophan metabolism. Tryptophan is metabolized into various AhR ligands. **a** In the gastrointestinal tract, various bacterial species in the microbiota can metabolize tryptophan to products with AhR agonistic effects. **b** Cruciferous vegetables produce the tryptophan metabolite glucosinolate via a hydrolysis reaction, yielding the AhR protoagonist I3C. In the stomach, I3C is metabolized by an acid-condensation reaction to AhR ligands 6-formylindolo(3,2-b)carbazole (FICZ), DIM and LTr1. **c** Host metabolites such as IS and IAA, with AhR agonistic effect, are primarily derived from tryptophan metabolism through the kynurenine pathway, with other ligands generated by ultraviolet exposure and oxidative reactions. B1: indole-3-acetaldehyde; B2: *S*-(indolylmethylthiohydroximoyl) l-cysteine; B3: indolylmethyl thiohydroximate; TPH: tryptophan hydroxylase

Declining renal function leads to the retention of various metabolites [41–45] that are retained in the blood and various tissues instead of being excreted by the kidneys [46]. Thus, the retention of these metabolites contributes to a variety of diseases, especially chronic kidney disease (CKD) and cardiovascular disease (CVD) [47–50]. CKD leads to the retention of one of the most important metabolites, so-called uremic solutes. In 2003, the European Uremic Toxin Work Group classified 90 uremic compounds [51]. The number of compounds/metabolites has since been extended [51]. Uremic toxins are classically categorized according to the physicochemical features affecting their clearance by dialysis: low water-soluble molecules (MW < 500 Da), larger middle molecules (MW > 500 Da) and protein-bound molecules [52]. Protein-bound uremic solutes are poorly removed through conventional dialysis. Among the uremic toxins, tryptophan-derived uremic toxins are of particular interest because they are implicated in cardiovascular toxicity and have been demonstrated to be potent AhR ligands [53, 54]. Tryptophan is an essential amino acid found in the diet. As shown in Fig. 3, 95% tryptophan can be metabolized via the kynurenine pathway, which is mediated by the rate-limiting enzymes tryptophan 2,3-dioxygenase (TDO) and indoleamine 2,3-dioxygenase (IDO) [55]. TDO is highly expressed in the liver. IDO has two isoenzymes, IDO1 and IDO2. IDO1 expression has been demonstrated in most tissues [55]. The activity of IDO leading from tryptophan to kynurenine is reflected by the tryptophan/kynurenine ratio [56]. Serum tryptophan is decreased in CKD patients, whereas metabolites from the kynurenine pathway, including kynurenine, kynurenic acid, 3-hydroxykynurenine, anthranilic acid and quinolinic acid, are increased. Two other tryptophan metabolic pathways are the serotonin pathway, which produces melatonin, and the indolic metabolic pathway, which produces indolic compounds, including indoxyl sulfate (IS), indole-3-acetic acid (IAA) and indoxyl-β-d glucuronide (IDG) (Fig. 3). In the indolic pathway, tryptophan is converted to indole through gut microbiota and absorbed into blood circulation [57] (Fig. 3). For instance, tryptophanase produced from *Escherichia coli* metabolizes dietary tryptophan to indole and its derivatives [58]. In the liver, bacterial-derived indole is further metabolized to IS via human *SULT1A1* [59]. Moreover, indole is oxidized to IS by microsomal *CYP2E1* [60]. IAA is directly produced in the gut by tryptophan metabolism or endogenously in tissue through tryptamine [60]. For example, tryptophan mono-oxygenase produced by *Arthrobacter pascens* and tryptophan decarboxylase produced by *Clostridium sporogenes* convert tryptophan into the AhR ligands IAA and tryptamine, respectively [61–63]. In the healthy state, the human gut microbiota carries out several activities to the body. Gut microbiota live in a commensal relationship with their host, protecting against pathogens, modulating the immune system, and regulating endogenous lipid and carbohydrate metabolism, thus maintaining the nutritional balance [64]. An increasing number of recent studies have demonstrated that alterations in gut microbiota are linked with a myriad of diseases, such as cancer, obesity, diabetes, cardiovascular disease, inflammatory bowel disease, and kidney disease [65]. It is increasingly recognized that gut microbiota metabolism contributes to the generation of enormous uremic toxins [66–69].

Although uremic toxins contribute to various diseases associated with a variety of action mechanisms, some metabolites, such as aromatic hydrocarbon metabolites and indole derivatives, have been demonstrated as endogenous AhR ligands and thus could evoke AhR activation [70]. Further study indicated that AhR seems to sense microbial insults and bacterial virulence factors, constituting a new AhR ligand [71]. Increasing evidence has also demonstrated that tryptophan metabolism-derived uremic toxins such as IS, IAA and IDG are recognized as the most important endogenous AhR ligands and thus can trigger AhR activation [72, 73] (Fig. 3). IS, IAA and IDG can activate AhR signalling via direct binding to the AhR/Hsp90/XAP2 complex. Both IS and IAA upregulate eight genes of AhR, including *CYP1A1* and *CYP1B1* [74].

IS has been reported as one of the most important uremic toxins. A panel of indole derivatives including tryptophan, indole, IS, IAA, and indole 3-methanol as AhR ligands has been examined [59]. IS has been demonstrated as a potent endogenous ligand that selectively activates human AhR at nanomolar level in primary human hepatocytes, mediating the transcription of multiple AhR genes, including *CYP1A1*, *CYP1A2*, *CYP1B1*, *UGT1A1*, *UGT1A6*, *interleukin 6* and *serum amyloid A1*. Furthermore, IS exerts a 500-fold greater potency in the transcriptional activation of human AhR compared to mouse AhR [59]. Structure–function findings have indicated that the sulfate group is an important factor for effective AhR activation. Ligand competition binding analyses have indicated that IS is a direct AhR ligand [59]. Previous studies have shown that IS suppresses endothelial proliferation, inhibits wound repair and triggers oxidative stress [72]. IS has been implicated in cardiovascular mortality and classical risk factors in CKD patients. Basal AhR level regulate podocyte function under normal circumstances, and upregulating AhR activation in podocytes by IS contributes to glomerular injury [75]. Activated AhR by IS triggers a proinflammatory phenotype, podocyte damage and glomerular injury both in vivo and in vitro [75]. Another study reported that increased IS affected iron metabolism in adenine-induced CKD mice by participating in hepcidin regulation via AhR and oxidative stress pathways [76]. Moreover, the activation of AhR mediated the IS-mediated upregulated expression of monocyte chemoattractant protein-1 (MCP-1) in human umbilical vein endothelial cells (HUVECs) [77]. IAA is another important uremic toxin. IAA activates the AhR/p38MAPK/NF-κB signalling pathway, which induces COX-2 expression, and IAA increases the production of reactive oxygen species both in vivo and in vitro [78]. Therefore, serum IS or IAA might be an independent indicator of cardiovascular events and mortality in CKD patients.

Other toxins from tryptophan metabolism reported as AhR ligands are from the kynurenine pathway. Kynurenic acid can induce the AhR signalling pathway in patients with CKD [79]. Moreover, 5/6 Nx rats exhibit high levels of serum kynurenine and 3-hydroxykynurenine and significantly upregulated AhR and *CYP1A1* mRNA expression in bone tissue compared to control rats [80]. Notably, the serum kynurenine level, the serum kynurenine/tryptophan ratio, and AhR and *CYP1A1* mRNA expression are lower in 3-month-old 5/6 Nx rats compared to 1-month-old 5/6 Nx rats [80].

### AhR activation mediates renal damage

#### The AhR pathway is activated in CKD

Patients with CKD are exposed to a high level of uremic toxins, causing an increased risk of cardiovascular disease. A number of uremic toxins, such as IS, IAA and IDG, are agonists of AhR. The latest study demonstrated that AhR was activated in patients with CKD stage 3 to 5D [81]. AhR-activating potential (AhR-AP) strongly correlates with eGFR and IS concentration. The expression of blood AHR target genes, including *CYP1A1* and *AhRR*, is upregulated in CKD patients compared to healthy controls [81]. Further study demonstrated that 5/6 nephrectomized (5/6 Nx) mice exhibited an increase in serum AhR-AP and an induction of *CYP1A1* mRNA expression in the heart and aorta that were absent in AhR^−/−^ CKD mice [81]. Increased serum AhR-AP level and upregulated *CYP1A1* mRNA level in the aortas and hearts from WT mice have been observed after serial IS injection, but not in AhR^−/−^ mice [81]. Taken together, these results suggest that the AhR signalling pathway is activated in both mice and patients with CKD.

Another study demonstrated that indolic uremic solutes upregulated tissue factor expression by an AhR-dependent pathway in patients with CKD (stages 3-5D) compared to healthy controls, evoking a ‘dioxin-like’ effect. Elevated tissue factors were positively correlated with serum IS and IAA concentrations in patients with CKD (stages 3-5D) [74]. In HUVECs, IS and IAA treatment further upregulated the expression of eight AhR genes: *CYP1A1*, *CYP1B1*, *CYP1A2*, *transforming growth factor β*-*3*, *prostaglandin G/H synthase and cyclooxygenase*, *CDD*-*inducible poly(ADP*-*ribose) polymerase*, *chemokine (C*–*C motif) receptor 7* and *AhRR*, the repressor of AhR [74]. The involvement of AhR activation in tissue factor production has been clarified by siRNA inhibition and with the AhR inhibitor geldanamycin [74]. These findings were amplified in peripheral blood mononuclear cells. The expression and activity of tissue factors were also augmented by TCDD. In addition, the IS level is significantly correlated with both AhR and tissue factor activities in patients with end-stage renal disease (ESRD) [82]. IS activates the AhR pathway in primary human aortic vascular smooth muscle cells, and AhR interacts directly with and stabilizes tissue factor. The AhR antagonist inhibits tissue factor, enhances tissue factor ubiquitination and degradation, and inhibits thrombosis and endovascular injury [82]. Moreover, monocytes respond to IS through AhR signalling and consequently upregulate tumour necrosis factor alpha expression in ESRD patients [83]. Taken together, these findings indicate that the activation of AhR is a key mechanism associated with deleterious cardiovascular disease in CKD.

However, the knockout of AhR leads to altered renal and hepatic phenotypes in both mice and rats [84]. AhR-knockout mice show changes in hepatic function and liver patent ductus venosus. AhR-knockout rats exhibit alterations in the urinary tract, including bilateral renal dilation (hydronephrosis), secondary tubular and uroepithelial degenerative alterations and bilateral ureter dilation (hydroureter) [84]. Another study indicated that aromatic hydrocarbons can compensate for renal cell function by intervening with mitochondrial function and glutathione homeostasis and are involved in both mesenchymal and epithelial populations in nephrotoxicity to this heterogeneous class of chemicals [85]. In addition, AhR stimulation can represent a novel renoprotective effect likely involving the mobilization and recruitment of Tregs and stem cells to the injured kidney [86].

The serum IS level is between 7 and 343 μM (mean value: 120–140 μM) in chronic haemodialysis patients [87]. IS circulates in albumin-bound and free forms. In haemodialysis patients, approximately 90% of IS is bound to serum proteins, indicating that the free serum IS level is 12 μM, an effective level that leads largely to the induction of AhR activity in cultured cells [88]. The IS level is highest in the kidney and lower in the lung, liver and heart in nephrectomized rats [89]. Rats with chronic renal failure showed sixfold higher IS level in kidney tissues and 71 μM IS in kidney homogenate. If these findings reflect the kidney tissue IS level in patients with ESRD, it would be expected that AhR could be fully activated and that AhR activation would further enhance the expression of AhR target genes, such as *CYP1A1*, *CYP1A2*, *CYP1B1* and *COX*-*2*. Another study indicated that CYP1A2 protein expression in the kidney and liver was greatly upregulated in rats with chronic renal failure [90]. In addition, TCDD can mediate hydronephrosis in mice [91]. TCDD toxicity is solely induced by AhR and thus could provide the clues for the inhibitory effects of elevated AhR activity mediated by high IS level.

Free IS, not albumin-bound IS, can activate AhR. One study investigated the effect of albumin-bound and free IS on AhR activation using IS level observed in different stages of patients with CKD. An AhR-driven reporter assay showed that both IS forms mediated dose-dependent AhR transcription in vascular smooth muscle cells [82]. IS level equivalent to those found in early stages of patients with CKD also increased AhR transcription, which was dose-dependently inhibited by the AhR antagonist CB7993113. Similarly, IS upregulated the expression of endogenous AhR target genes *CYP1A1*, *CYP1A2* and *AhRR*, all of which were abrogated by the AhR antagonist [82]. However, few studies have examined the effect of IS on AhR activity in renal proximal tubular cells. Taken together, these results show that upregulated AhR activity in the kidney may be associated with the high IS level in renal disease.

#### The AhR pathway is activated in diabetic nephropathy

Diabetic nephropathy has become a large global health problem. It has been reported that serum AhR transactivating activity is higher in type 2 diabetic patients with diabetic nephropathy with microalbuminuria, macroalbuminuria and ESRD than in subjects with normoalbuminuria [92]. Serum AhR ligands are correlated with the estimated glomerular filtration rate (eGFR), the serum creatinine level, systolic blood pressure, glycated haemoglobin and diabetic duration. High AhR transactivating activity is an independent risk factor in diabetic nephropathy [92]. A study on streptozotocin-induced diabetic mice showed that AhR deficiency reduced the induction of COX-2/prostaglandin E_2_, NADPH oxidase activity, oxidative stress, lipid peroxidation and *N*-Ɛ-carboxymethyllysine [93]. *N*-Ɛ-carboxymethyllysine significantly enhanced AhR/COX-2 DNA-binding activity, protein-DNA reciprocity, gene regulation, and ECM accumulation in renal proximal tubular cells and mesangial cells, which might be reversed by siRNA-AhR transfection [93]. In addition, human kidney dysfunction occurring as a result of diabetic nephropathy has been indicated to lead to high serum IS level [59].

#### AhR is associated with the RAS

The renin-angiotensin system (RAS) plays a key role in the progression of CKD. Several studies have indicated that AhR is associated with the RAS. For example, IS reduces the expression of the Mas receptor in the aortas of normotensive and hypertensive rats [94]. Another study demonstrated that IS downregulated the expression of the Mas receptor via AhR/NF-κB and mediated cell proliferation and tissue factor expression in human aortic smooth muscle cells. Ang-(1-7) suppressed IS-mediated tissue factor expression and cell proliferation by inhibiting phosphorylated ERK1/2 and NF-κB [94]. In addition, the expression of the Mas receptor is downregulated in the kidneys of rats with CKD [95]. IS induces the downregulation of the expression of the Mas receptor through the OAT3/AhR/STAT3 signalling pathway in proximal tubular cells [95]. The IS-mediated downregulation of the Mas receptor is involved in the upregulation of transforming growth factor beta 1 in proximal tubular cells. Another study indicated that IS induced the aortic expression of prorenin receptor and renin/prorenin via organic anion transporter 3-induced uptake, reactive oxygen species production and AhR and NF-κB p65 activation in vascular smooth muscle cells [96]. The IS-induced activation of the prorenin receptor promotes tissue factor expression and cell proliferation in vascular smooth muscle cells [96].

### The AhR pathway is activated in urinary system-associated cancers

The AhR pathway is involved in carcinogenesis [97]. It has been demonstrated that AhR is mainly expressed in the nuclei of advanced clear cell renal cell carcinoma (RCC) and tumour-infiltrating lymphocytes, and its expression correlates with the stage of the pathological tumour and the histological grade [98]. Matrix metalloproteinases (MMP) belong to a family of zinc-dependent endopeptidases and are considered as therapeutic targets for renal diseases [99]. AhR activation upregulates the mRNA expression of its target genes *CYP1A1* and *CYP1B1* and promotes invasion by upregulating the mRNA expression of *MMP*-*1*, *MMP*-*2* and *MMP*-*9* and downregulating the mRNA expression of E-cadherin in human RCC cell lines, including 786-O and ACHN [98]. Furthermore, a siRNA for AhR downregulated CYPs and inhibited cancer cell invasion accompanied by the downregulation of MMP in 786-O cells [98]. These findings indicate that AhR regulates cell RCC invasion involved in tumour immunity. The same study group showed that nuclear AhR expression was also significantly related to pathological T stage, histological grade, invasion and lymph node involvement in patients with upper urinary tract urothelial carcinoma [100]. AhR expression is considered an independent predictor of disease-specific survival. T24 UC cells induced by TCDD showed the upregulated mRNA expression of AhR, *CYP1A1* and *CYP1B1* accompanied by the upregulated mRNA expression of *MMP*-*1* and *MMP*-*9* and enhanced T24 cell invasion [100]. Furthermore, T24 cells transfected with a siRNA for AhR showed the downregulated mRNA expression of *AhR*, *CYP1A1*, *CYP1B1*, *MMP*-*1*, *MMP*-*2* and *MMP*-*9* and indicated decreased invasion ability [100]. Taken together, these findings indicate that AhR plays an important role in the invasiveness of cancer cells and can serve as a prognostic biomarker and potential therapeutic target for patients with urinary system-associated cancers.

### Natural products such as AhR agonists or antagonists in kidney disease and renal cell carcinoma

Organic anion transporting polypeptides and organic anion transporters play a key role in renal uremic toxin elimination. Solute carrier organic anion transporter family member 4C1 (SLCO4C1) is the only organic anion transporting polypeptide expressed at the basolateral side of human renal proximal tubular cells, and it modulates uremic toxin excretion. Human SLCO4C1 overexpression in rat kidneys promotes renal uremic toxin excretion and lowers cardiomegaly, hypertension and renal inflammation in renal failure [101]. Statin induces SLCO4C1 expression via AhR by binding to the XRE at its promoter region [101]. Statin administration promotes the elimination of uremic toxins and mitigates organ damage in a rat renal failure model. MicroRNAs play an important role in the cellular defence mechanism. It has been reported that miR-125b is transcriptionally activated by Nrf2 and could be an inhibitor of the AhR repressor in cisplatin-induced mice, which contributes to protecting the kidney from acute kidney injury [102].

Natural products in the clinic have been regarded as an alternative therapy for the prevention and treatment of a myriad of diseases worldwide [103–107]. Natural products also continue to provide a protean and unique source of new bioactive lead candidates for drug discovery [108–115]. Numerous studies have demonstrated a variety of natural product-derived compounds that can directly activate or inhibit AhR [116–119]. As early as the 1970s, several studies reported that the ligands of AhR from vegetable extracts or vegetable-derived materials mediate *CYP1A1* activity [120, 121]. As shown in Fig. 3, members of the cruciferous family, such as broccoli, brussels sprouts, white cabbage and cauliflower, have rich sources of glucobrassicin or glucosinolate conjugates that produce indole-3-carbinol (I3C) and indole-3-acetonitrile (I3AC) by using enzymatic cleavage during mastication [122, 123]. I3C and I3AC can bind to and activate AhR. Indolo[3,2,-b]carbazole (ICZ) and 3,3′-diindolylmethane (DIM) are two major acidic condensation products of I3C. ICZ has a higher affinity for the AhR ligand compared to other natural products [124]. 3,3′-Diindolylmethane is an established AhR agonist [124]. Glucosinolate conjugates can activate AhR in mice and humans [121, 125]. After consumption, glucosinolates undergo hydrolysis, transferring I3C, ICZ, DIM and ([2-(indol-3-ylmethyl)-indol-3-yl] indol-3-ylmethane (LTr1), which serve as AhR agonists [121]. These compounds are involved in gut AhR expression needed for the maintenance of innate lymphoid cells and intraepithelial lymphocytes. These findings demonstrate an important link between dietary factors, AhR and intestinal immunity. Subsequently, studies demonstrated that a number of compounds from natural products, such as I3C, curcumin, quercetin, resveratrol, 7,8-dihydrorutacarpine, dibenzoylmethanes and carotinoids (canthaxanthin, astaxanthin and β-apo-8′-carotenal), could competitively bind to AhR and/or mediate the expression of AhR-dependent genes [116, 117, 123, 126].

Polyphenols are widespread compounds throughout the plant kingdom [127]. They are characterized by using a classic phenol ring chemical structure. According to the phenol ring amounts in compounds and the approach they use, polyphenols are divided into 5 categories: flavonoids, phenolic acids, stilbenes, lignans and tannins [128]. Flavonoids and phenolic acids are the most abundant polyphenols in the daily diet and can be further divided into several categories based on the oxidation degree of the oxygen heterocycle, including flavonols, flavanols, flavanones, flavones, isoflavones, proanthocyanidins and anthocyanins [128]. Flavonoids from natural products constitute the largest category of AhR ligands [117, 129, 130]. Flavonoids, such as kaempferol, (–)-epigallocatechin gallate, luteolin, myricetin, epigallocatechin, morin, galangin, eriodictyol, tangeritin, apigenin and naringenin, are mostly AhR antagonists, but some of them, including chrysin, baicalein, quercetin, diosmin, icaritin, tangeritin, and tamarixetin, are AhR agonists [116, 117, 126]. In addition to interplaying with AhR, many flavonoids are also substrates for *CYP1A1*. These flavonoids are widely distributed in medicinal plants, fruits, vegetables and teas, and flavonoid concentrations in human blood are in the low micromolar range, levels sufficient to inhibit/activate AhR [116]. Thus, it is not surprising that the extracts of many natural products exhibit AhR agonist and/or antagonist activity. Therefore, natural products commonly include AhR ligands or natural products that can be transformed into AhR ligands, and as such, flavonoids are the largest class of natural AhR ligands that are available for humans and animals. Mounting evidence has demonstrated that polyphenols, especially flavonoids, as modulators of AhR, are widely used for the regulation of the intestinal immune system and tumour treatment [116–118, 131, 132], but only several studies have reported that natural products regulate AhR in kidney damage.

Aristolochic acids, such as aristolochic acid I (AAI) and aristolochic acid II, with the structure of nitrophenanthrene carboxylic acids, are the main active component of *Aristolochia* species [133]. Aristolochic acids were known to possess anti-inflammatory properties until the first case of nephropathy was found in Belgium, which is now regarded as aristolochic acid nephropathy (AAN) [134]. AA exposure was recently implicated in Balkan endemic nephropathy and associated with urothelial cancer [135, 136]. The mechanism revealed that AAI-mediated nephrotoxicity is associated with deficient liver-specific NADPH-cytochrome P450 reductase, and the induction of *CYP1A* significantly lowers AAI-induced kidney toxicity [134]. Baicalin significantly alleviates AAI-mediated kidney toxicity via AhR-dependent *CYP1A1* and *CYP1A2* induction in the liver [137]. Tanshinone I promotes AAI metabolism and prevents AAI-mediated kidney injury by the induction of hepatic *CYP1A1* and *CYP1A2* in vivo [138].

## Concluding remarks

Initially, AhR was discovered as a chemical-sensing signalling molecule that mediated the toxic responses from environmental pollutants. In recent years, increasing studies on AhR ligands have testified an unparalleled expansion from exogenous toxic responses to many biology- and medicine-related fields, such as cancer, immunity regulation, cardiovascular disease and kidney disease. AhR signalling exhibits a variety of biological functions that have expanded its classical transcriptional function into the regulation of the cytosolic signalling pathway and possesses novel endogenous ligands that can bind to and activate AhR-dependent gene expression. Although the recently reported AhR ligands have expanded greatly, many related studies of AhR are still rigorously challenging, and great effort should be made in the future.

First, the structural identification of AhR ligands could provide insight into novel exogenous and endogenous ligands of AhR. Although fractionation approaches of biological samples could not identify many endogenous ligands in the past, the latest development of high-throughput, rapid and sensitive metabolomic approaches provides avenues for the identification, isolation, and characteristics of new AhR ligands from trace amounts of complex matrices and biological samples. Metabolomics and lipidomics have been successfully utilized to discover and identify a variety of novel endogenous AhR ligands, especially aromatic hydrocarbon-containing metabolites (uremic toxins), in both animal models and patients with CKD [50, 139–143]. Overall, characterization of the spectrum of endogenous AhR ligands will provide novel molecular and biochemical mechanisms by which ligands can induce AhR activation.

Second, natural products have been extensively used for the prevention and intervention of a myriad of diseases worldwide. Investigators revealed an intriguing trend in drug development beginning in the 21st century: a return to nature as a source of novel potential agents [144, 145]. Natural products possess a wide range of bioactivities and were a continuous source of novel drug leads that contributed to approximately 46% of drugs approved by the Food and Drug Administration from 1981 to 2014 [146–149]. The abovementioned studies have demonstrated that flavonoids are AhR antagonists or agonists. Flavonoids are widely distributed in natural products such as medicinal plants, fruits, vegetables and teas. To date, more than 15,000 flavonoids have been identified from natural products [150]. Due to their importance in the regulation of AhR activity, great effort should be made to further investigate the regulation of flavonoids on AhR activity. A better understanding of their chemical structures and AhR biological activity will be of importance to uncover their further potential as therapeutic drugs and their molecular mechanism.

Third, uremic toxins from tryptophan metabolism and dioxins from environmental pollutants activate the AhR signalling pathway. These toxins induce leukocyte activation and endothelial dysfunction, causing thrombosis and inflammation as well as enhanced vascular oxidative stress. Uremic toxins from tryptophan metabolism that activate AhR explain how these toxins contribute to CVD in CKD patients. These mechanisms of toxicity of uremic toxins may provide new potential therapeutic approaches targeting AhR activation. Although a number of experiments have explored the relationship between AhR activity and various kidney diseases by analysing the target genes of AhR in both animal models and patients with CKD, AhR in kidney disease is still in its infancy compared with cancer and immune disease. A large number of metabolites, especially uremic toxins, have been identified by high-throughput metabolomics, although the number is apparently insufficient. Further studies should be performed on the effect of novel metabolites on AhR activity. In addition, the AhR pathway can interact with the Wnt/β-catenin, transforming growth factor-β/bone morphogenetic protein and Notch signalling pathways as well as tyrosine kinase receptor pathways, including vascular endothelial growth factor receptor, keratinocyte growth factor receptor and epidermal growth factor receptor, in several human diseases [118]. Many studies have well documented that the transforming growth factor-β/bone morphogenetic protein, Wnt/β-catenin, Notch signalling and tyrosine kinase receptor pathways are involved in CKD [151, 152]. Few studies have demonstrated whether AhR can interact with these signalling pathways in kidney disease.

Finally, regardless of the promising translational and clinical applications of AhR, most of the knowledge currently available about its physiopathological function has been demonstrated by using animal models, which have led to certain limitations for the direct transfer of achievements into patients. Great effort will surely focus on the validation of data from animal experiments to clinical application in the future, and system biology, including genomics, transcriptomics, proteomics, metabolomics and lipidomics, will most likely play an important role in studies on AhR. These are exciting areas for future studies. It is very likely that future studies will provide new diagnostic and prognostic approaches for complex human diseases and may establish new therapeutic strategies targeting AhR activation.

## Data Availability

Not applicable.

## Abbreviations

5/6 Nx: 5/6 nephrectomized
AAI: aristolochic acid I
AAN: aristolochic acid nephropathy
AhR: aryl hydrocarbon receptor
AhR-AP: AhR-activating potential
AhRR: AhR repressor
ARNT: AhR nuclear translocator
CKD: chronic kidney disease
COX-2: cyclooxygenase-2
CVD: cardiovascular disease
CYP1A1: cytochrome P450, family 1, member 1A
CYP1A2: cytochrome P450, family 1, member 2A
CYP1B1: cytochrome P450, family 1, sub family B
DIM: diindolylmethane
DRE or XRE: dioxin- or xenobiotic-responsive element
eGFR: estimated glomerular filtration rate
ERK: extracellular signal-regulated kinase
ESRD: end-stage renal disease
HSP90: heat-shock protein 90
HUVECs: human umbilical vein endothelial cells
I3AC: indole-3-acetonitrile
I3C: indole-3-carbinol
IAA: indole-3-acetic acid
ICZ: indolo[3,2,-b]carbazole
IDG: indoxyl-β-d-glucuronide
IDO: indoleamine 2,3-dioxygenase
IS: indoxyl sulfate
JNK: jun-NH2-terminal kinase
LTr1: [2-indol-3-ylmethyl:-indol-3-yl] indol-3-ylmethane
MAPK: mitogen-activated protein kinase
MCP-1: monocyte chemoattractant protein-1
MMP: metalloproteinase
NF-κB: nuclear factor kappa B
Nrf2: nuclear factor-erythroid-2-related factor 2
P23: co-chaperon p23
Per-ARNT-Sim: period-aryl hydrocarbon receptor nuclear translocator-single minded
RAS: renin-angiotensin system
RCC: renal cell carcinoma
SLCO4C1: solute carrier organic anion transporter family member 4C1
SULT: sulfotransferase
TCDD: 2,3,7,8-tetrachlorodibenzo-*p*-dioxin
TDO: tryptophan 2,3-dioxygenase
XAP2: X-associated protein 2
